# Integrative regulation of middle cortex formation: from classic modules to emerging pathways

**DOI:** 10.3389/fpls.2025.1705659

**Published:** 2025-11-10

**Authors:** Seung Woo Kim, Kwang Suk Chang, Minhee Kang, Jun Lim

**Affiliations:** Department of Systems Biotechnology, Konkuk University, Seoul, Republic of Korea

**Keywords:** *Arabidopsis*, gibberellic acid, GRAS transcription factor, ground tissue, middle cortex formation, root development

## Abstract

Generation of distinct cell types through asymmetric cell division (ACD) is a fundamental developmental process in multicellular organisms. Therefore, controlling when and where ACDs occur is essential for the production of new cells and tissues. The *Arabidopsis* (*Arabidopsis thaliana*) root has emerged as a powerful model for studying this process because its cell division patterns are highly stereotyped and easily observed. Within the ground tissue, periclinal ACDs in the endodermis generate the middle cortex (MC) post-embryonically, which serves as a hallmark of root maturation. Since the first description of MC formation, extensive research has identified the genetic and environmental cues that either promote or suppress its initiation. Over the past two decades, studies have revealed that MC formation is orchestrated by a regulatory hub centered on the SHORT-ROOT (SHR)–SCARECROW (SCR) transcriptional module and its target, CYCLIND6;1 (CYCD6;1). This core pathway is fine-tuned by multiple regulators, including transcriptional co-activators, repressors, and integrators of gibberellic acid (GA) signaling. Recent advances have uncovered new roles for transcription factors, chromatin regulators, redox enzymes, and receptor-like kinases in linking hormonal signals and positional cues to the SHR–SCR–CYCD6;1 regulatory hub. Together, these pathways ensure that MC formation occurs at the right time, place, and extent. This review summarizes advances in MC regulation, highlighting how transcriptional, hormonal, and positional networks integrate to ensure developmental plasticity in plant roots.

## 1 Introduction

Asymmetric cell division (ACD) is a universal developmental mechanism operating across multicellular organisms and plays a fundamental role in cell fate determination, tissue patterning, and stem cell maintenance (Horvitz and Herskowitz, 1992; Knoblich, 2008; Ten Hove and Heidstra, 2008; Abrash and Bergmann, 2009; De Smet and Beeckman, 2011). Through ACD, a single progenitor cell gives rise to two daughter cells with distinct identities, enabling cellular diversification. Consequently, ACD is considered a key developmental innovation underlying multicellularity. Because spatial and temporal regulation of ACD determines tissues organization, elucidating when, where, and how ACD occurs remains a central question in developmental biology.

**Figure 1:**
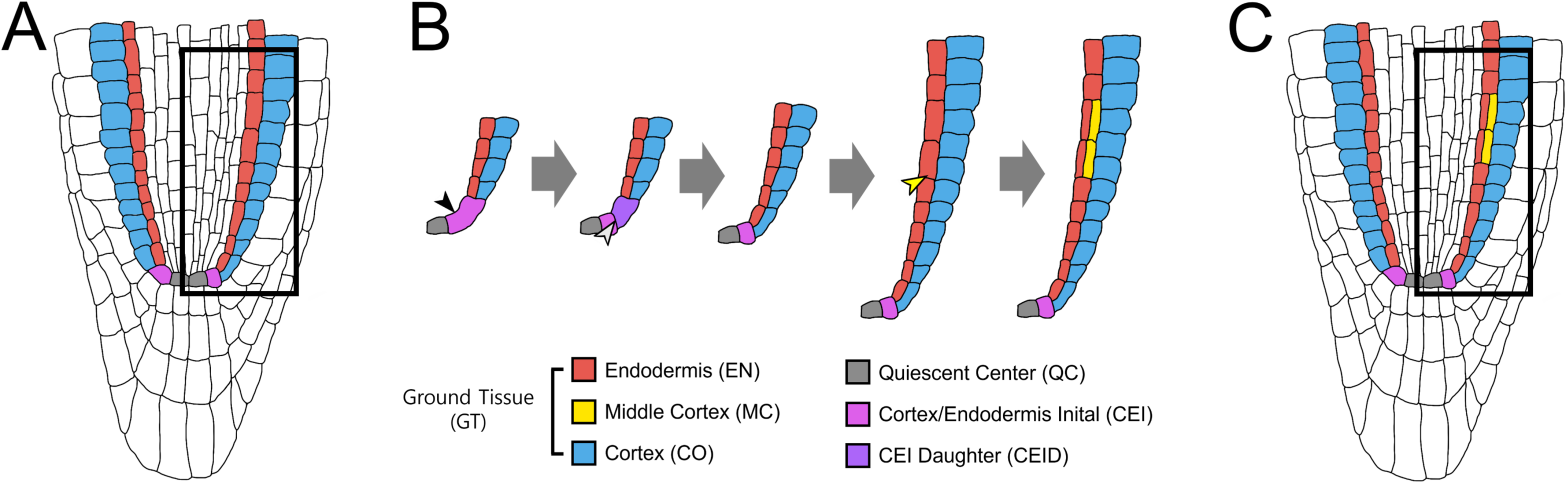
Ground tissue patterning and MC formation in the *Arabidopsis* root. **(A)** Organization of the *Arabidopsis* root meristem. The quiescent center (QC) and the surrounding stem cells establish the stem cell niche, which generates distinct root cell lineages. **(B)** Lineage of the ground tissue layers. The cortex/endodermis initial (CEI) undergoes an anticlinal asymmetric cell division (ACD) to self-renew and generate a CEI daughter (CEID). Subsequent periclinal ACD of the CEID produces one cortical layer (CO, blue) and one endodermal layer (EN, red), establishing the radial ground tissue pattern. During post-embryonic maturation, endodermal cells undergo additional periclinal ACDs, giving rise to the middle cortex (MC, yellow) positioned between cortex and endodermis. **(C)** Mature root ground tissue. At maturity, the ground tissue is composed of three layers: endodermis (EN), middle cortex (MC), and cortex (CO) (from inside to outside).

The *Arabidopsis* (*Arabidopsis thaliana*) root serves as an ideal model to study ACD regulation due to stereotyped organization and optical accessibility, allowing direct lineage tracing (Schiefelbein and Benfey, 1991; Benfey et al., 1993; Dolan et al., 1993; Scheres et al., 1994, 1995; Benfey and Scheres, 2000; Benfey et al., 2010). Within the root meristem, cortex/endodermis initials (CEIs) undergo anticlinal ACDs to renew itself and produce a CEI daughter (CEID). Subsequent periclinal ACDs of CEIDs generate the endodermis and cortex (Benfey et al., 1993; Dolan et al., 1993; Scheres et al., 1994, 1995; Benfey and Scheres, 2000; Di Ruocco et al., 2018) (**Figures 1A, B**).

The genetic basis of ground tissue patterning was first revealed through *short-root* (*shr*) and *scarecrow* (*scr*) mutants, which disrupt cortex–endodermis organization (Benfey et al., 1993; Scheres et al., 1995; Di Laurenzio et al., 1996; Helariutta et al., 2000). *shr* lacks an endodermis, while *scr* forms a single mixed cortex–endodermis layer. A striking feature of post-embryonic ground tissue patterning is the formation of the middle cortex (MC), an additional cortical layer generated by periclinal ACDs in the endodermis (Baum et al., 2002) (**Figures 1B, C**). Unlike the periclinal ACDs of CEID that establish the initial cortex–endodermis separation, MC formation occurs later and serves as a hallmark of ground tissue maturation (Baum et al., 2002; Paquette and Benfey, 2005; Cui and Benfey, 2009a, 2009b; Pauluzzi et al., 2012; Petricka et al., 2012; Cui, 2015, 2016; Choi and Lim, 2016). Notably, in addition to formative ACDs for the cortex–endodermis split, SHR and SCR play key roles in endodermal ACDs during MC formation. For instance, *scr* precociously forms MC layers with high frequency (Paquette and Benfey, 2005), suggesting that SCR exerts spatiotemporal control over periclinal ACDs, promoting early endodermis–cortex separation whereas restricting later MC initiation. In contrast, *shr* lacks both endodermis and MC, underscoring the necessity of a functional endodermis for MC production (Paquette and Benfey, 2005).

Since the initial description of MC formation (Baum et al., 2002), extensive studies have identified the genetic and environmental cues that modulate formative ACDs in the endodermis. In particular, SHR and SCR activity is tuned by developmental timing and by interactions with additional regulators, including cell cycle genes and hormonal pathways, ensuring that MC formation occurs only at the appropriate developmental stage (Paquette and Benfey, 2005; Cui and Benfey, 2009a, 2009b; Heo et al., 2011; Koizumi et al., 2012a, 2012b; Cui et al., 2014; Wu et al., 2014; Gong et al., 2016; Lee et al., 2016; Zhang et al., 2018; Campos et al., 2020; Bertolotti et al., 2021; Tian et al., 2022; Oh et al., 2023; Xie et al., 2023; Chang et al., 2024). Thus, the SHR–SCR module serves as a central component of the genetic circuitry underlying MC production by integrating positional information with the control of cell division and fate specification. MC formation provides a powerful framework for understanding how transcriptional networks and hormonal pathways converge to regulate the timing and extent of ACDs in multicellular development. Importantly, the SHR–SCR module is closely linked to the gibberellic acid (GA) pathway, forming a central regulatory hub that coordinates intrinsic transcriptional programs with extrinsic hormonal cues (Paquette and Benfey, 2005; Cui and Benfey, 2009a, 2009b; Heo et al., 2011; Cui et al., 2014; Gong et al., 2016; Lee et al., 2016; Bertolotti et al., 2021; Oh et al., 2023; Chang et al., 2024).

In this review, we highlight the recent advances in elucidating how the SHR–SCR–GA regulatory hub, along with its associated transcriptional and receptor-like kinase (RLK) modules, orchestrates the precise spatiotemporal control of formative ACDs for MC production.

## 2 The SHR–SCR–CYD6;1 core module in MC formation

The SHR–SCR complex represents the central transcriptional unit governing root ground tissue patterning. SHR, a GRAS-domain transcription factor expressed in the stele, moves into the adjacent cell layers (e.g., endodermis), where it is retained in the nucleus by SCR, another GRAS transcription factor (Di Laurenzio et al., 1996; Pysh et al., 1999; Helariutta et al., 2000; Nakajima et al., 2001; Gallagher et al., 2004; Cui et al., 2007; Gallagher and Benfey, 2009). Together, they form a stable transcriptional complex, which has been shown to regulate ground tissue patterning throughout development (Di Laurenzio et al., 1996; Helariutta et al., 2000; Wysocka-Diller et al., 2000; Cui et al., 2007; Cruz-Ramírez et al., 2012; Koizumi et al., 2012a, 2012b; Hirano et al., 2017; Long et al., 2017; Hernández-Coronado and Ortiz-Ramírez, 2021).

A key downstream target of the SHR–SCR module is *CYCLIND6;1* (*CYCD6;1*), which encodes a D-type cyclin required for formative ACDs in the *Arabidopsis* root (Sozzani et al., 2010). *CYCD6;1* is expressed spatiotemporally in CEI and CEID cells, coinciding with the periclinal ACDs that generate the cortex and endodermis. Its expression reappears later in endodermal cells undergoing formative ACDs during MC formation. Consistently, *cycd6;1* mutants display reduced periclinal ACDs with delayed and infrequent MC initiation, confirming its essential role (Sozzani et al., 2010).

Interestingly, SCR negatively regulates MC formation by repressing *CYCD6;1* expression, as evidenced by the precocious MC development observed in *scr* (Paquette and Benfey, 2005; Heo et al., 2011). SHR also functions in a dose-dependent manner: high SHR levels suppress MC initiation, whereas intermediate levels promote it (Koizumi et al., 2012b). Notably, low threshold levels of SHR and SCR are sufficient to act early in the cell cycle, determining the orientation of the division plane and thereby directing formative versus proliferative divisions in root stem cells (Winter et al., 2024).

Although SHR and SCR evidently regulate *CYCD6;1* transcription, the connection to the general transcriptional machinery long remained unresolved. Zhang et al. (2018) provided mechanistic insight by showing that the Mediator subunit MED31 directly interacts with SCR (but not SHR). MED31 enables Pol II recruitment to the *CYCD6;1* promoter, ensuring timely activation. Through SCR as an interface protein, MED31, SCR, and SHR assemble into a dynamic ternary complex, in which MED31 and SHR compete for binding to SCR. Reduction of MED31 (*MED31-RNAi*) disrupted this regulation: *CYCD6;1* expression was lost in CEI/CEID cells but ectopically activated in upper ground tissue cells, leading to irregular periclinal ACDs and supernumerary cell layers (Zhang et al., 2018). These findings establish MED31 as a critical co-activator that couples the SHR–SCR developmental module to the Pol II machinery, ensuring the precise spatiotemporal activation of *CYCD6;1* for endodermal ACDs during MC formation.

In contrast to CEI/CEID cells, where *CYCD6;1* must be activated to drive formative ACDs, its expression in upper ground tissue cells must be repressed to prevent excessive MC production. The mechanisms underlying this negative regulation of *CYCD6;1* were recently clarified by Xie et al. (2023), who identified NAC1 (NAM, ATAF1/2, and CUC2 domain transcription factor 1) as a direct repressor of *CYCD6;1*. Loss-of-function *nac1* mutants display excessive periclinal ACDs in the endodermis, thereby causing an earlier and higher frequency MC production. Conversely, *NAC1* overexpression suppresses MC formation. Mechanistically, NAC1 binds directly to the *CYCD6;1* promoter at the NAC recognition motifs and recruits the co-repressor TOPLESS (TPL) to repress transcription. NAC1 also interacts with SCR and SHR, and its occupancy at the *CYCD6;1* promoter depends on SCR. Importantly, NAC1 antagonizes SHR by displacing it from the *CYCD6;1* promoter, thereby limiting SHR-mediated activation (Xie et al., 2023). These findings demonstrate that NAC1 fine-tunes root ground tissue patterning by repressing *CYCD6;1* in an SCR-dependent manner, antagonizing SHR activity, and preventing MC overproduction.

Taken together, the SHR–SCR module acts as a finely tuned developmental rheostat, rather than a simple on–off switch, ensuring that ACDs occur with the appropriate frequency and orientation. This regulatory flexibility provides developmental plasticity, enabling roots to adjust ground tissue patterning in response to intrinsic transcriptional programs and extrinsic environmental signals.

## 3 The SHR–SCR–CYD6;1 module in GA-mediated MC formation

Classic work by Paquette and Benfey (2005) demonstrated that GA levels strongly influence the timing of formative ACDs during MC formation. Under GA-deficient conditions, either genetically (e.g., the *ga1-3* loss-of-function mutant impaired in GA biosynthesis) or pharmacologically (treatment with the GA biosynthesis inhibitor paclobutrazol, PAC), endodermal cells undergo periclinal ACDs earlier and more frequently. Conversely, the exogenous application of bioactive GAs suppresses these divisions, thereby restricting or delaying MC production (Paquette and Benfey, 2005; Heo et al., 2011). These findings highlight the importance of precise modulation of GA levels in regulating MC formation within the root ground tissue.

The GA pathway is tightly integrated with the SHR–SCR transcriptional module, which provides the competence for endodermal ACDs. While SHR–SCR activity defines the spatial and developmental windows for periclinal divisions, GA signaling fine-tunes their timing and frequency. This interplay ensures that MC initiation is coordinated with overall root growth and developmental progression.

### 3.1 The downstream and upstream regulatory networks of SHR–SCR–CYCD6 in GA-mediated MC formation

A pivotal mediator of the crosstalk between the SHR–SCR module and GA signaling is SCARECROW-LIKE 3 (SCL3), a GRAS transcription factor that integrates developmental and hormonal pathways in the endodermis (Heo et al., 2011). SCL3 is a direct target of the SHR–SCR complex and functions downstream of the core module. By antagonizing DELLA repressors, SCL3 modulates GA responses, ensuring a balanced signaling output (Heo et al., 2011; Zhang et al., 2011). Loss-of-function *scl3* mutants disrupt this balance, resulting in mistimed or excessive MC divisions, whereas proper SCL3 activity confers developmental robustness under fluctuating hormonal or environmental conditions. Thus, SCL3 acts as an endodermis-specific integrator of transcriptional and hormonal cues, reinforcing the model in which GA restricts premature MC formation by acting synergistically with the SHR–SCR module.

Recent work has extended SCL3’s role beyond GA signaling to the regulation of reactive oxygen species (ROS) homeostasis during MC formation. Oh et al. (2023) identified PRX34, a class III peroxidase, as a downstream component that produces hydrogen peroxide (H_2_O_2_), a positive regulator of periclinal ACDs. GA-deficient conditions elevate H_2_O_2_ levels, thereby accelerating MC initiation. Consistently, *prx34* mutants show reduced H_2_O_2_ accumulation and diminished MC formation, whereas exogenous H_2_O_2_ rescues the phenotype. Genetic analysis placed *PRX34* downstream of *SCL3*: *prx34 scl3* double mutants resembled *prx34*, indicating that SCL3 negatively regulates PRX34 to fine-tune H_2_O_2_ levels (Oh et al., 2023). Through this regulation, SCL3 integrates GA signaling and ROS homeostasis, preventing excessive ROS accumulation and ensuring robust control of MC production during ground tissue maturation (Oh et al., 2023).

In parallel, SPINDLY (SPY), which encodes an *O*-fucosyltransferase that activates DELLA proteins (Zentella et al., 2017), regulates numerous genes involved in oxidative stress and redox homeostasis, including peroxidases (e.g., PRX34). The *spy* mutant exhibits elevated H_2_O_2_ levels, leading to premature MC formation (Cui et al., 2014). Consistently, antioxidant treatment with glutathione suppresses MC production in *spy* mutants, indicating that, similar to PRX34, SPY contributes to the maintenance of cellular redox balance. Although both SPY and PRX34 participate in ROS regulation, no direct genetic or biochemical interaction between them has been reported to date. Given that SPY functions as a negative regulator of GA signaling, and that GA influences ROS dynamics through DELLA-dependent mechanisms, it is plausible that SPY may affect PRX34 activity indirectly through GA–ROS crosstalk.

Bertolotti et al. (2021) revealed that the HD-ZIP III transcription factors PHABULOSA (PHB) and PHAVOLUTA (PHV) are also crucial for GA-mediated MC formation. *phb phv* double mutants delay MC initiation, whereas the gain-of-function *phb-1d* allele induces precocious and excessive MC production. PHB acts non-cell-autonomously from the vasculature to activate *CYCD6;1* expression in the endodermis. In parallel, PHB modulates the stability of DELLA proteins (notably GAI) through transcriptional activation of *GA2OX2*, which encodes a GA catabolic enzyme (Bertolotti et al., 2021). By reducing bioactive GA levels, PHB stabilizes DELLA repressors, which in turn promote *CYCD6;1* accumulation in the endodermis, thereby stimulating MC initiation. Thus, PHB coordinates cell cycle activation and hormone metabolism, linking vascular regulation with endodermal patterning. This PHB-dependent pathway likely functions in concert with the SHR–SCR–SCL3 hub to ensure balanced GA activity and DELLA function during MC formation.

Upstream of the SHR–SCR–SCL3 hub, SEUSS (SEU) functions as a GA-responsive activator (Gong et al., 2016). Loss-of-function *seu* mutants exhibit early and frequent MC divisions, whereas *SEU* overexpression markedly reduces them, highlighting SEU as a negative regulator of formative ACDs during MC formation. SEU directly binds to the promoters of *SHR*, *SCR*, and *SCL3*, establishing its role as a direct upstream regulator in the ground tissue. Interestingly, *SEU* expression itself is GA-responsive: repressed by exogenous GA but induced under GA-deficient conditions. Moreover, GA fails to repress *SCL3* expression in the *seu* background, indicating that SEU is essential for the GA-mediated transcriptional control (Gong et al., 2016). By linking hormonal cues to transcriptional regulation, SEU operates in a feedback loop that balances GA signaling with SHR–SCR activity, ensuring the correct timing and extent of MC formation.

### 3.2 The positional regulation downstream of SHR–SCR–CYCD6 in MC formation

Cell polarity is crucial for controlling cell division orientation and tissue patterning. Two leucine-rich repeat RLKs, INFLORESCENCE AND ROOT APICES RECEPTOR KINASE (IRK) and KINASE ON THE INSIDE (KOIN), act downstream of the SHR–SCR transcriptional network to repress cell division and maintain proper root patterning (Campos et al., 2020; Rodriguez-Furlan et al., 2022). The two receptors exhibit opposite polar localization: KOIN resides at the inner (stele-facing) plasma membrane, whereas IRK localizes to the outer (cortex-facing) domain of endodermal cells. Of them, IRK functions to repress *CYCD6;1* expression, thereby preventing premature or ectopic periclinal ACDs (Campos et al., 2020). In *irk* mutants, elevated *CYCD6;1* expression triggers early and excessive MC formation. By negatively regulating *CYCD6;1*, IRK counterbalances the SHR–SCR core module, establishing a dual system that integrates transcriptional activation and polarity-dependent repression of formative divisions. Thus, the endodermis serves as a regulatory hub, using polarized receptors to perceive and coordinate bidirectional developmental signals, thereby ensuring the proper spatial organization of the root tissue. In this framework, the polarized localization of IRK translates positional information into cell division control, providing a mechanistic link between membrane polarity and developmental patterning.

Recent work by Chang et al. (2024) further identified three RLKs (ARH1, FEI1, and FEI2) as novel regulators of MC formation. Triple RLK mutants (*tri-1* and *tri-2*) exhibit excessive MC layers due to reduced GA biosynthesis, resulting in stabilized DELLA proteins and expanded *CYCD6;1* expression. Exogenous GA application or mutation of *CYCD6;1* suppresses this phenotype, confirming their role in the GA–DELLA–CYCD6;1 regulatory network. These RLKs act mainly cell-autonomously in the endodermis and are negatively regulated by SHR and SCR. Moreover, the loss of *RLK*s partially rescues *shr* and *scr* defects, placing them downstream of the SHR–SCR module but upstream of GA biosynthesis. Although direct binding of SHR or SCR to the *RLK* promoters has not been detected, these kinases represent a mechanistic bridge linking transcriptional programs to hormone metabolism (Chang et al., 2024).

Collectively, these findings define a multilayered SHR–SCR–CYCD6;1 hub, in which transcription factors, receptor kinases, and hormonal pathways integrate to precisely control the timing, spatial pattern, and frequency of endodermal ACDs for MC formation.

## 4 Concluding remarks and prospects

**Figure 2:**
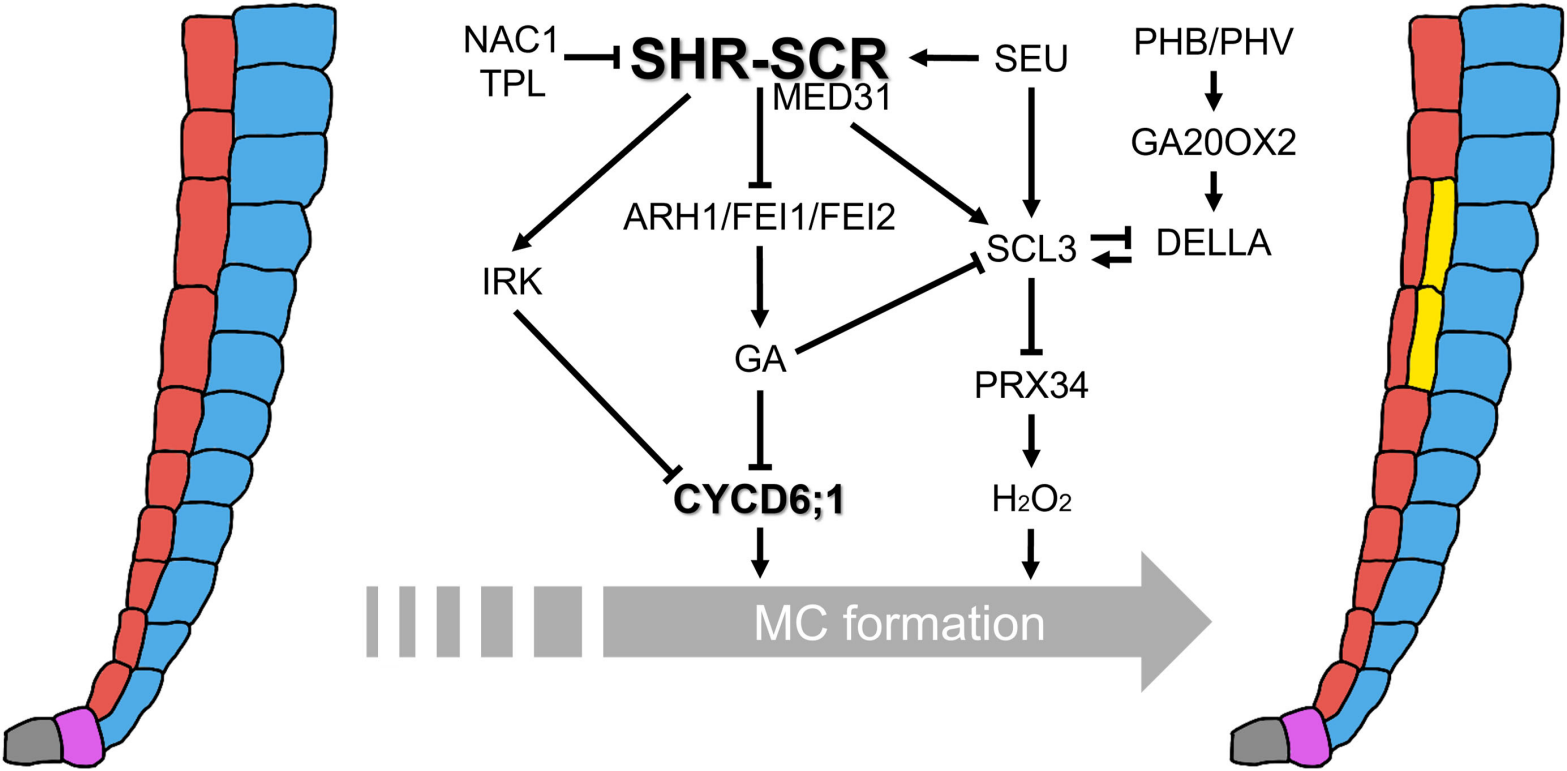
Integrative regulatory networks controlling MC formation. The central regulatory hub is the SHR–SCR transcriptional complex, which directly activates *CYCD6;1* expression and confers competence to periclinal ACDs in the endodermis. Multiple interconnected pathways modulate this activity: MED31 couples the SHR–SCR complex to Pol II to ensure proper *CYCD6;1* activation; NAC1, together with the co-repressor TPL, represses *CYCD6;1* by antagonizing SHR–SCR function; SEU acts as a GA-responsive activator of *SHR*, *SCR*, and *SCL3*; PHB/PHV promote *CYCD6;1* non-cell-autonomously from the vasculature and stabilize DELLA proteins through transcriptional activation of *GA2OX2*. GA signaling delays MC initiation by destabilizing DELLAs, whereas SCL3 buffers GA responses and represses *PRX34*, thereby fine-tuning hydrogen peroxide (H_2_O_2_) levels that promote periclinal ACDs. RLKs (ARH1, FEI1, FEI2, and IRK) function downstream of the SHR–SCR module but upstream of GA biosynthesis. Their loss reduces GA levels, stabilizes DELLAs, and expands *CYCD6;1* expression. Together, these multilayered networks integrate transcriptional, hormonal, redox, and positional cues to ensure that MC formation occurs with the proper timing, spatial precision, and frequency.

MC formation in the *Arabidopsis* root exemplifies how transcriptional programs, hormonal signals, and positional cues converge to regulate formative ACDs. At the core of this process lies the SHR–SCR–CYCD6;1 module, whose activity is fine-tuned by transcription factors (e.g., SCL3, PHB, SEU, and NAC1), receptor kinases (e.g., IRK and ARH1/FEI1/FEI2), and GA signaling (**Figure 2**). In addition to GA, other hormones, including abscisic acid, auxin, brassinosteroids, ethylene, and salicylic acid, also modulate MC initiation (Choi and Lim, 2016; Lee et al., 2016; Pasternak et al., 2019; Li et al., 2020; Seo et al., 2021; Tian et al., 2022; Rawat and Laxmi, 2025). Collectively, these pathways establish a multihormone regulatory landscape that dynamically modulates the SHR–SCR–CYCD6;1 network to ensure robust and context-dependent ground tissue patterning.

The SHR–SCR complex represents a conserved regulatory module mediating ACDs for radial patterning across plant lineages. Nevertheless, it has been revealed that through gene duplication, diversification of expression domains, and acquisition of novel interaction partners, the SHR–SCR module has been repurposed to regulate diverse ground tissue patterning programs in both roots and shoots (reviewed in Di Ruocco et al., 2018; Shaar-Moshe and Brady, 2023). This underscores the evolutionary flexibility and modularity of the SHR–SCR network, which bridges conserved developmental mechanisms with evolutionary innovation in plant tissue organization.

Despite these advances, several fundamental questions remain. How are diverse hormonal inputs integrated at the SHR–SCR hub to produce coherent developmental outputs? How do polarity cues provided by RLKs interact with transcriptional and hormonal regulation? To what extent are these regulatory mechanisms conserved or diversified in species with more complex root architectures?

Addressing these questions will require methodological innovations. Single-cell transcriptomics can provide high-resolution maps of cell identity and hormone responsiveness, while live imaging of hormone and ROS dynamics will allow spatiotemporal tracking of regulatory events. Coupled with targeted genetic perturbations, these approaches will illuminate how regulatory networks operate in real time and under variable environmental conditions.

Ultimately, dissecting the intersection of transcriptional regulators, hormonal signals, and positional cues in MC formation will not only deepen our understanding of developmental plasticity but also offer strategies to engineer root systems with enhanced adaptability to environmental challenges—a priority in the context of global climate change and agricultural sustainability.
